# Assessment of paravalvular regurgitation after transcatheter aortic valve replacement using 2D multi-velocity encoding and 4D flow cardiac magnetic resonance

**DOI:** 10.1093/ehjci/jeae035

**Published:** 2024-02-02

**Authors:** Maxim J P Rooijakkers, Saloua El Messaoudi, Niels A Stens, Marleen H van Wely, Jesse Habets, Monique Brink, Laura Rodwell, Daniel Giese, Rob J van der Geest, Niels van Royen, Robin Nijveldt

**Affiliations:** Department of Cardiology, Radboud University Medical Centre, Geert Grooteplein Zuid 10, 6525 GA, Nijmegen, The Netherlands; Department of Cardiology, Radboud University Medical Centre, Geert Grooteplein Zuid 10, 6525 GA, Nijmegen, The Netherlands; Department of Cardiology, Radboud University Medical Centre, Geert Grooteplein Zuid 10, 6525 GA, Nijmegen, The Netherlands; Department of Medical BioSciences, Radboud University Medical Centre, Nijmegen, The Netherlands; Department of Cardiology, Radboud University Medical Centre, Geert Grooteplein Zuid 10, 6525 GA, Nijmegen, The Netherlands; Department of Radiology and Nuclear Medicine, Radboud University Medical Centre, Nijmegen, The Netherlands; Department of Radiology and Nuclear Medicine, Haaglanden Medical Centre, The Hague, The Netherlands; Department of Radiology and Nuclear Medicine, Radboud University Medical Centre, Nijmegen, The Netherlands; Department of Health Sciences, Section Biostatistics, Radboud Institute for Health Sciences, Nijmegen, The Netherlands; Magnetic Resonance, Siemens Healthcare GmbH, Erlangen, Germany; Institute of Radiology, Friedrich-Alexander-Universität Erlangen-Nürnberg (FAU), University Hospital Erlangen, Erlangen, Germany; Department of Medical Imaging, Leiden University Medical Centre, Leiden, The Netherlands; Department of Cardiology, Radboud University Medical Centre, Geert Grooteplein Zuid 10, 6525 GA, Nijmegen, The Netherlands; Department of Cardiology, Radboud University Medical Centre, Geert Grooteplein Zuid 10, 6525 GA, Nijmegen, The Netherlands

**Keywords:** aortic stenosis, cardiac magnetic resonance, paravalvular regurgitation, transcatheter aortic valve replacement

## Abstract

**Aims:**

To compare the novel 2D multi-velocity encoding (venc) and 4D flow acquisitions with the standard 2D flow acquisition for the assessment of paravalvular regurgitation (PVR) after transcatheter aortic valve replacement (TAVR) using cardiac magnetic resonance (CMR)-derived regurgitant fraction (RF).

**Methods and results:**

In this prospective study, patients underwent CMR 1 month after TAVR for the assessment of PVR, for which 2D multi-venc and 4D flow were used, in addition to standard 2D flow. Scatterplots and Bland–Altman plots were used to assess correlation and visualize agreement between techniques. Reproducibility of measurements was assessed with intraclass correlation coefficients. The study included 21 patients (mean age ± SD 80 ± 5 years, 9 men). The mean RF was 11.7 ± 10.0% when standard 2D flow was used, 10.6 ± 7.0% when 2D multi-venc flow was used, and 9.6 ± 7.3% when 4D flow was used. There was a very strong correlation between the RFs assessed with 2D multi-venc and standard 2D flow (*r* = 0.88, *P* < 0.001), and a strong correlation between the RFs assessed with 4D flow and standard 2D flow (*r* = 0.74, *P* < 0.001). Bland–Altman plots revealed no substantial bias between the RFs (2D multi-venc: 1.3%; 4D flow: 0.3%). Intra-observer and inter-observer reproducibility for 2D multi-venc flow were 0.98 and 0.97, respectively, and 0.92 and 0.90 for 4D flow, respectively.

**Conclusion:**

Two-dimensional multi-venc and 4D flow produce an accurate quantification of PVR after TAVR. The fast acquisition of the 2D multi-venc sequence and the free-breathing acquisition with retrospective plane selection of the 4D flow sequence provide useful advantages in clinical practice, especially in the frail TAVR population.

## Graphical Abstract

**Graphical Abstract:**
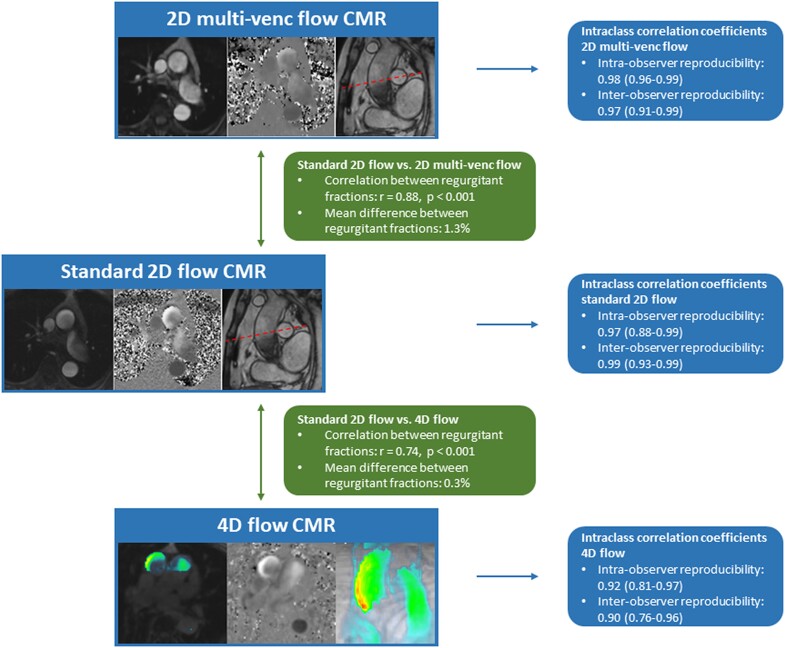
Assessment of paravalvular regurgitation after transcatheter aortic valve replacement using novel CMR flow assessment techniques. CMR, cardiac magnetic resonance; venc, velocity encoding.

## Introduction

Aortic stenosis (AS) is the most common valvular heart disease in the Western world. Randomized trials have demonstrated non-inferiority or superiority of transcatheter aortic valve replacement (TAVR) compared with surgical aortic valve replacement in patients with severe AS across the spectrum of surgical risk.^1–6^ An important shortcoming of TAVR is the relatively high risk for the development of paravalvular regurgitation (PVR). The occurrence of PVR is common after TAVR, with incidences of mild PVR up to 40% and incidences of ≥moderate PVR up to 10% in contemporary TAVR studies.^7,8^ In patients with ≥moderate PVR, mortality is three times higher compared with patients with none to trace PVR.^9–11^ Recently, increasing evidence suggests that mild-to-moderate or even only mild PVR could also have an impact on mortality.^12,13^ Given the strong implications for post-TAVR survival, early identification of relevant PVR is warranted to guide additional interventions (e.g. post-dilation) in order to reduce PVR and improve patient outcomes.

Although echocardiography remains the cornerstone of valvular heart disease assessment, visualization, and specifically quantification of aortic regurgitation after TAVR are challenging. Quantitative flow assessment using 2D through-plane phase-contrast (PC) cardiac magnetic resonance (CMR) has less inter-observer variability than echocardiography,^14^ is not limited by its acoustic windows secondary to patient characteristics (e.g. obesity and chronic obstructive pulmonary disease [COPD]), and allows unlimited imaging plane selection. Emerging techniques in the field of flow assessment after TAVR using CMR are 2D multi-velocity encoding (venc) flow mapping and 4D flow mapping. Two-dimensional multi-venc flow mapping facilitates the use of a single breath-hold to analyse two or three different venc values by combining these individual venc values into a single reconstruction that can be used for flow quantification.^15^ In 4D flow mapping, 3D blood flow patterns and haemodynamics can be assessed along all three spatial dimensions and over the complete cardiac cycle.^16^ Four-dimensional flow assessment is not dependent on breath-holds, thus allowing the patient to breathe freely during acquisition. As a result, no breathing-dependent variation is observed. Moreover, since the analysis plane can be set anywhere within the acquisition volume offline, 4D flow mapping is less operator dependent.^17^

The aim of this substudy of the Assessment of Paravalvular Regurgitation After Transcatheter Aortic Valve Replacement by Haemodynamic Measurements and Cardiac Magnetic Resonance (APPOSE) trial was to assess the validity of the 2D multi-venc and 4D flow mapping techniques in PVR quantification when compared with the standard 2D flow mapping technique.

## Methods

### Population and design

The APPOSE trial (NCT04281771) was an investigator-initiated, prospective, single-centre study evaluating the accuracy of haemodynamic indices to predict relevant PVR as quantified by CMR.^18^ In short, patients were eligible for the study if they underwent TAVR for severe symptomatic AS, with main exclusion criteria of a pre-existing cardiac device, left ventricular ejection fraction (LVEF) <30%, and a serum creatinine >250 µmol/L or end-stage renal disease. In all patients, a self-expanding valve (Portico; Abbot Structural Heart, Minneapolis, MN, USA) was implanted, with valve sizes ranging between 23 and 29 mm. Pre-TAVR echocardiographic assessment included aortic valve area, mean and peak aortic valve gradients, and LVEF. Between November 2019 and October 2021, 103 patients provided written informed consent for the APPOSE trial. A total of 77 patients were included in the primary analysis.

Patients were eligible for the present substudy if they underwent evaluation of PVR by CMR using the 2D multi-venc and/or 4D flow mapping sequence, in addition to the standard 2D flow mapping acquisition. This substudy enrolled 21 consecutive patients between March 2021 and October 2021, upon the installation of both 2D multi-venc and 4D flow mapping sequences to the CMR system. The study protocol was approved by the local Medical Research Ethics Committee and by the institutional review board of the Radboud University Medical Centre. The APPOSE trial was funded by a research grant from Abbott.

### Echocardiographic assessment of PVR

Transthoracic echocardiography (TTE) to determine the degree of PVR was performed 4–6 weeks after TAVR. Echocardiographic grading of PVR was based on an integrative multi-parametric approach that included a visual assessment of the number of PVR jets, jet width at the origin, and the circumferential extent of PVR. The degree of PVR was classified into none/trace, mild, moderate, or severe, according to Valve Academic Research Consortium-3 criteria.^19^ Two researchers (M.J.P.R. and S.E.M.) independently assessed the echocardiographic degree of PVR. If consensus was not reached, a third researcher got involved (R.N.).

### CMR acquisition

All patients were scanned on the same day as the TTE assessment (4–6 weeks after TAVR) on a commercially available 1.5-T CMR scanner (MAGNETOM Avanto; Siemens Healthcare, Erlangen, Germany).

#### Standard 2D flow mapping

Using a 2D PC velocity-encoded spoiled gradient echo pulse sequence, the slice for the through-plane velocity quantification was placed perpendicular to the direction of the flow 5 mm above the most cranial part of the TAVR bioprosthesis, so that there were no metal artefacts of the struts in the 2D image. Standard 2D flow mapping acquisitions were performed during successive end-expiratory breath-holds. One cardiac cycle consisted of 25 phases. A high venc of ≥180 cm/s was used for accurate assessment of the forward volume (FV). In case of aliasing, the venc was increased with subsequent steps of 20 cm/s until no more aliasing was observed. The assessment of whether or not aliasing was present was done by two observers (M.J.P.R. and a CMR laboratory technician). A low venc of 75 cm/s was used for the determination of the regurgitant volume (RV), in order to acquire accurate measurements of diastolic flow. Regurgitant fraction (RF) was calculated by dividing the RV (low venc) by the FV (high venc), multiplied by 100. Standard 2D flow mapping was considered the gold standard in PVR assessment in this study.

#### Two-dimensional multi-venc flow mapping

A research sequence was applied for the 2D multi-venc flow mapping acquisition. The same acquisition plane was used as for the standard 2D flow mapping. Three different venc values were used to obtain a single flow curve. These three different venc values were the following (in ascending order): a low venc of 75 cm/s (typical cut-off for low-flow assessment, e.g. pulmonary flow), the same venc that was used during standard 2D flow mapping in which no aliasing was observed (i.e. ≥180 cm/s), and a high venc of 300 cm/s (typical cut-off for high-flow assessment, e.g. moderate AS). Based on a Bayesian unfolding algorithm,^20^ a single velocity image per cardiac frame was reconstructed and used for post-processing.^21,22^ The 2D multi-venc acquisition was performed in a single end-expiratory breath-hold.

#### Four-dimensional flow mapping

A research sequence was applied for the 4D flow mapping acquisition during free breathing. With a field-of-view extending from the left ventricular outflow tract to the ascending aorta, the venc at which no aliasing was observed during 2D flow (i.e. ≥180 cm/s) was used. For breathing motion compensation, a navigator was placed onto the liver–diaphragm border, and respiration was tracked at every electrocardiogram cycle. Only data acquired during expiration were accepted. In order to decrease scan time, an under-sampling factor of 8 was used, and data were reconstructed using compressed sensing.^23,24^

### Image analysis

CMR post-processing was performed using Medis Suite MR software (version 4.0.24.4, Medis Medical Imaging, Leiden, The Netherlands). QFlow version 8.1 was used for the analysis of the standard 2D flow and 2D multi-venc mapping acquisitions. QFlow 4D version 1.1 was used for the analysis of the 4D flow mapping acquisition. The region of interest was placed in the ascending aorta 5 mm above the most cranial part of the TAVR bioprosthesis, as indicated previously.

Background phase correction was routinely applied for the standard 2D flow and 4D flow mapping sequences. Background phase was not observed in the static tissue of the 2D multi-venc reconstructed images; therefore, no background phase correction was applied. This can be attributed to the reconstruction algorithm, which chooses the optimal velocity value of the three single-venc acquisitions and averages out the background phases of the individual single-venc images.^21^

Two researchers (M.J.P.R. and N.A.S.) independently analysed the CMR data, which was supervised by a third researcher (R.N., >10 years of experience in cardiac imaging). The researchers were blinded for both the CMR data and the echocardiographic data.

### Statistical analysis

Data are presented as mean ± standard deviation or number (percentage), as appropriate. Pearson’s correlation coefficients were used to quantify the association between continuous variables. Agreement between the different flow assessment techniques was visually assessed by performing a Bland–Altman analysis.^25^ Reclassification of PVR severity when applying the novel CMR techniques was visualized through a Sankey diagram. Reproducibility of standard 2D flow, 2D multi-venc flow, and 4D flow mapping measurements was evaluated by using intraclass correlation coefficients (ICCs). ICCs for absolute agreement of single measures were estimated using a two-way random effects model to estimate the inter-observer reproducibility, whereas a two-way mixed effects model was used to estimate the intra-observer reproducibility. Intra-observer assessment was performed in a blinded fashion between initial and repeat measurements, with a time period of 2 weeks between the assessments. The inter-observer assessment was also performed with blinding for the other observer’s measurements. All statistical tests were two tailed, and a *P*-value of <0.05 was considered statistically significant. Analyses were performed in SPSS Statistics (version 27.0.1.0, IBM Corporation, Armonk, NY, USA).

## Results

### Baseline characteristics

Baseline characteristics of the 21 patients are presented in *Table 1*. The mean age was 79.8 ± 4.9 years, and 42.9% of patients were men. The mean European System for Cardiac Operative Risk Evaluation (EuroSCORE) II was 2.62 ± 1.32, with 57.1% of patients being in New York Heart Association (NYHA) function Class III or IV. Baseline echocardiographic parameters were as follows: the mean LVEF was 53.3 ± 9.3%, with a mean aortic valve area and aortic valve mean gradient of 0.81 ± 0.19 cm^2^ and 45.7 ± 6.4 mmHg, respectively.

**Table 1:** Baseline characteristics

	Study population (*N* = 21)
**Demographics**	
Age, years	79.8 ± 4.9
Male sex, *n* (%)	9 (42.9)
Body mass index, kg/m^2^	28.0 ± 3.7
Obesity, *n* (%)	5 (23.8)
**Medical history**	
EuroSCORE II	2.62 ± 1.32
NYHA Class III/IV, *n* (%)	12 (57.1)
Diabetes mellitus, *n* (%)	7 (33.3)
Coronary artery disease, *n* (%)	13 (61.9)
COPD, *n* (%)	3 (14.3)
Atrial fibrillation, *n* (%)	4 (19.0)
**Pre-procedural echocardiographic parameters**	
Aortic valve area, cm^2^	0.81 ± 0.19
Aortic valve mean gradient, mmHg	45.7 ± 6.4
Aortic valve maximum velocity, m/s	4.3 ± 0.3
LVEF, %	53.3 ± 9.3
Moderate or severe aortic regurgitation, *n* (%)	2 (9.5)

Data are presented as mean ± standard deviation or as number (%).

COPD, chronic obstructive pulmonary disease; EuroSCORE, European System for Cardiac Operative Risk Evaluation; LVEF, left ventricular ejection fraction; NYHA, New York Heart Association.

Due to the extensiveness of the CMR scan protocol, in which the 2D multi-venc and 4D flow mapping sequences were performed at the end of the scanning procedure, four patients failed to undergo the full scan protocol. Hence, there were two patients with missing 2D multi-venc data and two patients with missing 4D flow mapping data. In the remaining 17 patients, all 3 different flow sequences were successfully acquired.

### Echocardiographic assessment of PVR

The mean duration between TAVR and TTE was 38 ± 11 days. TTE assessment showed none/trace PVR in 14 (66.7%) patients, mild PVR in 7 (33.3%) patients, and no patients with ≥moderate PVR.

### CMR quantification of RF

The CMR assessment of PVR is provided in *Table 2*.

**Table 2:** CMR and echocardiographic assessment of PVR

	Study population
Days after TAVR	38 ± 11
**2D flow measurements (*N* = 21)**	
FV, mL	83.5 ± 19.6
RV, mL	9.8 ± 7.9
RF, %	11.7 ± 10.0
Classification of PVR	
Mild or less than mild (RF ≤ 20%), *n* (%)	18 (85.7)
Moderate (RF 21–39%), *n* (%)	2 (9.5)
Severe (RF ≥ 40%), *n* (%)	1 (4.8)
**2D multi-venc flow measurements (*N* = 19)**	
FV, mL	73.6 ± 17.2
RV, mL	7.7 ± 4.9
RF, %	10.6 ± 7.0
Classification of PVR	
Mild or less than mild (RF ≤ 20%), *n* (%)	17 (89.5)
Moderate (RF 21–39%), *n* (%)	2 (10.5)
Severe (RF ≥ 40%), *n* (%)	0
**4D flow measurements (*N* = 19)**	
FV, mL	71.7 ± 14.6
RV, mL	7.0 ± 5.7
RF, %	9.6 ± 7.3
Classification of PVR	
Mild or less than mild (RF ≤ 20%), *n* (%)	16 (84.2)
Moderate (RF 21–39%), *n* (%)	3 (15.8)
Severe (RF ≥ 40%), *n* (%)	0
**TTE classification of PVR** ^a^	
None/trace, *n* (%)	14 (66.7)
Mild, *n* (%)	7 (33.3)
Moderate, *n* (%)	0
Severe, *n* (%)	0

Data are presented as mean ± standard deviation or as number (%).

^a^TTE was performed on the same day as CMR.

FV, forward volume; PVR, paravalvular regurgitation; RF, regurgitant fraction; RV, regurgitant volume; TAVR, transcatheter aortic valve replacement; TTE, transthoracic echocardiography; venc, velocity encoding.

#### Standard 2D flow mapping

Standard 2D flow assessment was done in 21 patients. The mean FV measured with a high venc (≥180 cm/s) was 83.5 ± 19.6 mL. The mean RV measured with a low venc (75 cm/s) was 9.8 ± 7.9 mL, resulting in a mean RF of 11.7 ± 10.0%. The mean acquisition times of the high and low venc sequences were 15.0 ± 2.2 and 15.0 ± 2.0 s, respectively. Compared with standard 2D flow mapping assessment, TTE underestimated the degree of PVR in 3 of 21 patients.

#### Two-dimensional multi-venc flow mapping

In 19 patients, 2D multi-venc flow mapping measurements were performed. The mean FV and RV were 73.6 ± 17.2 and 7.7 ± 4.9 mL, respectively, yielding a mean RF of 10.6 ± 7.0%. The mean acquisition time was 10.7 ± 1.4 s. Compared with 2D multi-venc flow mapping assessment, TTE underestimated the degree of PVR in 2 of 19 patients.

#### Four-dimensional flow mapping

Four-dimensional flow assessment was done in 19 patients. The mean FV and RV were 71.7 ± 14.6 and 7.0 ± 5.7 mL, respectively, resulting in a mean RF of 9.6 ± 7.3%. The mean acquisition time was 184.6 ± 37.7 s. Compared with 4D flow assessment, TTE underestimated the degree of PVR in 2 of 19 patients.

### Correlation between CMR flow assessment techniques

**Figure 1:**
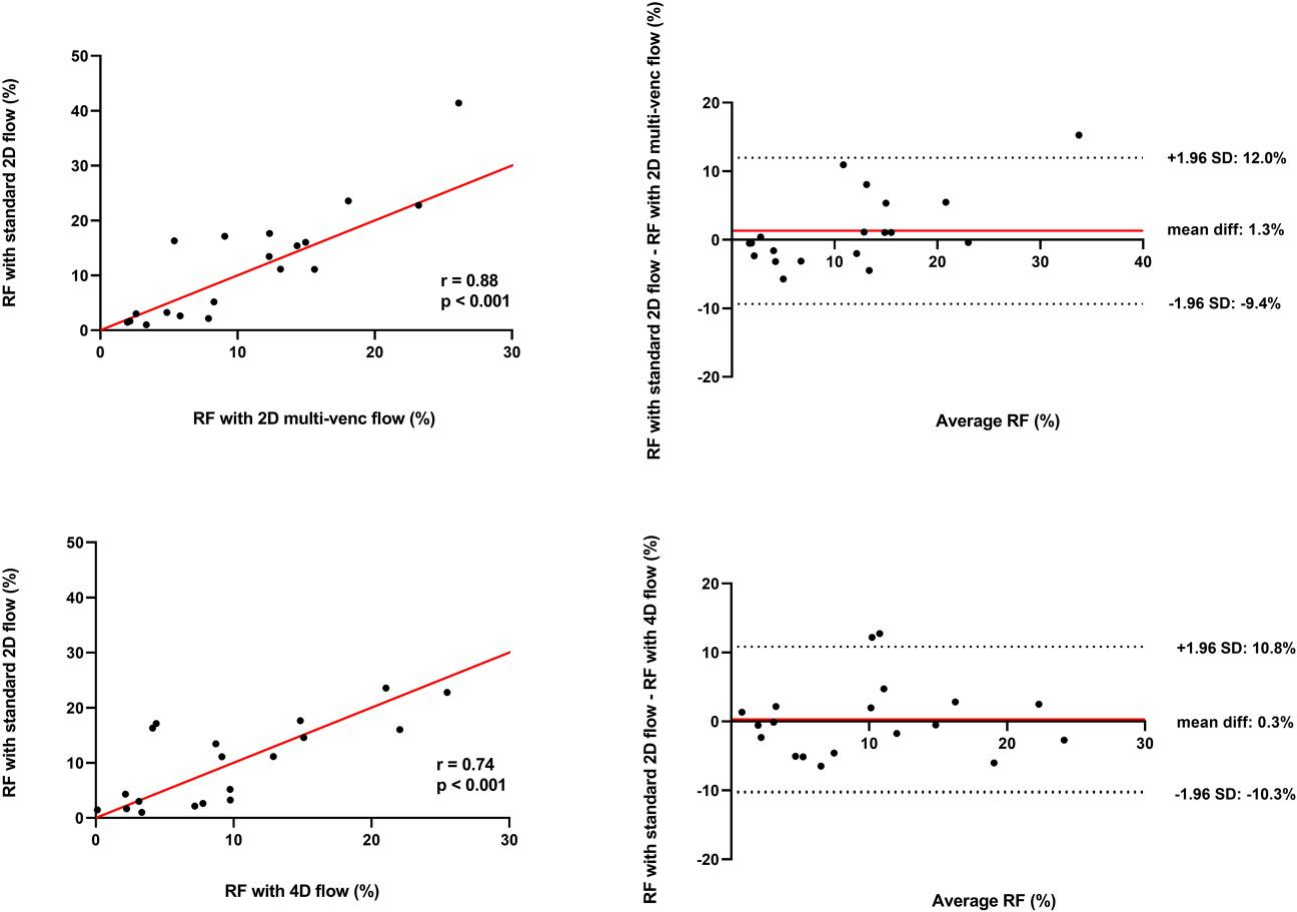
Relationship between different flow assessment techniques. Scatter (left) and Bland–Altman (right) plots demonstrating the relationship between RF assessed with standard 2D flow and 2D multi-venc flow mapping (top) and standard 2D flow and 4D flow mapping (bottom). RF, regurgitant fraction; SD, standard deviation; venc, velocity encoding.

Correlation analysis showed that RF measured with 2D multi-venc flow mapping was strongly correlated with RF as assessed with standard 2D flow mapping (*r* = 0.88, *P* < 0.001; *Figure 1*). Bland–Altman analysis demonstrated a mean bias of 1.3 ± 5.4% between the values of RF quantified with 2D multi-venc vs. RF quantified with standard 2D flow mapping. RF measured with 4D flow mapping had a good correlation with RF as assessed with standard 2D flow (*r* = 0.74, *P* < 0.001). Bland–Altman analysis demonstrated a mean bias of 0.3 ± 5.4% between the values of RF quantified with 4D flow mapping vs. RF quantified with standard 2D flow mapping.

**Figure 2:**
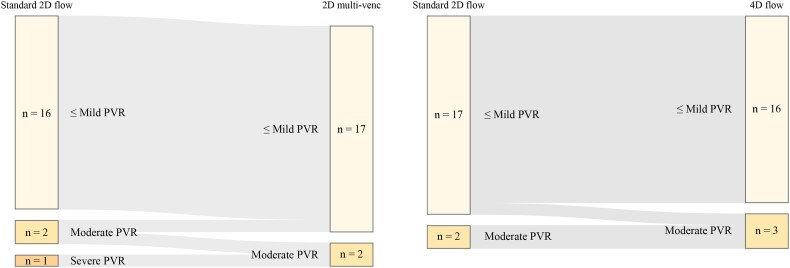
Reclassification of PVR severity between the different flow assessment techniques. Sankey diagram visualizing the reclassification of PVR severity between standard 2D flow and 2D multi-venc flow mapping (left) and standard 2D flow and 4D flow mapping (right). PVR, paravalvular regurgitation; venc, velocity encoding.

Reclassification of PVR severity based on standard 2D flow measurements on the one hand and 2D multi-venc or 4D flow measurements on the other hand are displayed in *Figure 2*.

Two-dimensional multi-venc reclassified one patient with severe PVR (according to standard 2D flow) into moderate PVR and one patient with moderate PVR into mild PVR. All other patients were classified into the same category of PVR severity. Four-dimensional flow reclassified one patient with mild PVR (according to standard 2D flow) into moderate PVR, while all other patients were classified into the same category of PVR severity.

### Reproducibility of standard 2D, 2D multi-venc, and 4D flow measurements

ICCs for intra-observer and inter-observer reproducibility of standard 2D, 2D multi-venc, and 4D flow mapping measurements are presented in *Table 3*.

**Table 3:** ICCs of standard 2D flow, 2D multi-venc flow, and 4D flow mapping

	Intra-observer	Inter-observer
Standard 2D flow	0.97 (0.88–0.99)	0.99 (0.93–0.99)
2D multi-venc flow	0.98 (0.96–0.99)	0.97 (0.91–0.99)
4D flow	0.92 (0.81–0.97)	0.90 (0.76–0.96)

Data are ICCs (95% confidence interval).

ICC, intraclass correlation coefficient; venc, velocity encoding.

**Figure 3:**
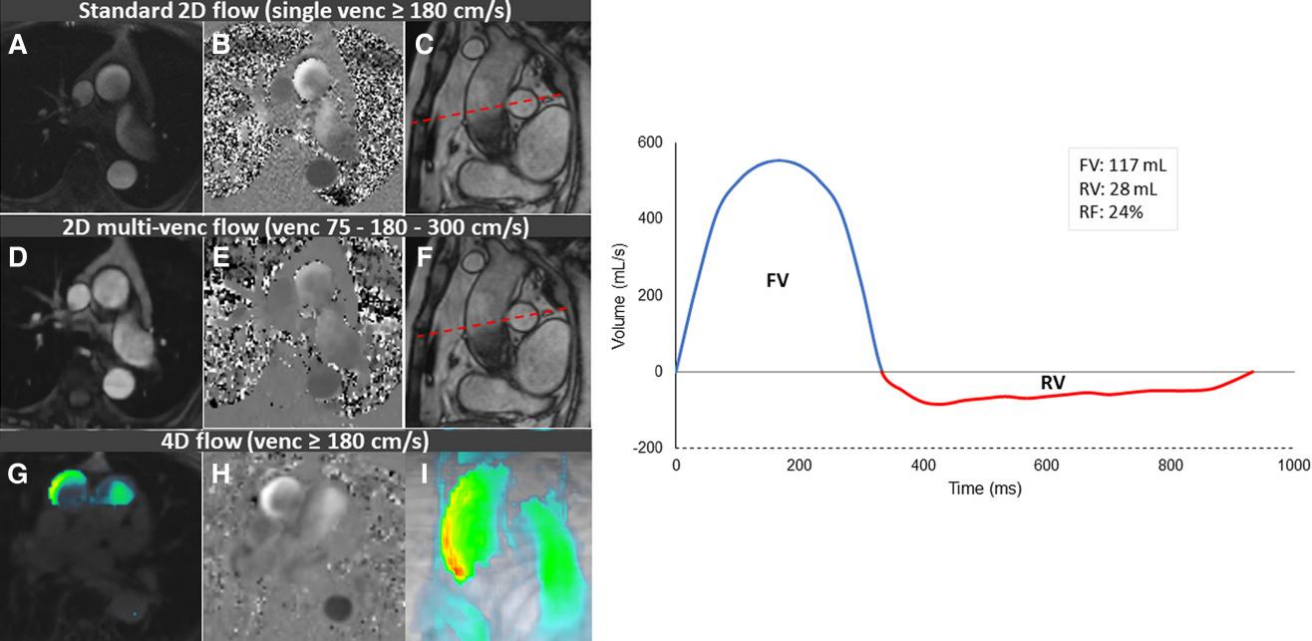
Example of the three different CMR flow assessment techniques. Left: upper row: standard 2D flow mapping with (*A*) magnitude image; (*B*) phase image; (*C*) the level of acquisition of PC CMR indicated with the dashed line. Middle row: 2D multi-venc flow mapping with (*D*) magnitude image; (*E*) phase image; (*F*) the level of acquisition of PC CMR indicated with the dashed line. Lower row: 4D flow mapping with (*G*) magnitude image; (*H*) phase image; and (*I*) 4D flow image reconstruction. Right: the quantification of the RF was done by dividing the RV by the FV multiplied by 100. FV, forward volume; RF, regurgitant fraction; RV, regurgitant volume; venc, velocity encoding.

An example of the three different CMR flow assessment techniques can be seen in *Figure 3*.

## Discussion

In this prospective non-selective cohort study in consecutive patients undergoing TAVR, we investigated the correlation between RF as assessed with 2D multi-venc and 4D flow imaging, and RF as assessed with standard 2D flow imaging.

The key findings can be summarized as follows. First, we found that both 2D multi-venc and 4D flow mapping were strongly correlated with standard 2D flow mapping regarding the measurement of RF (*r* = 0.88, *P* < 0.001 and *r* = 0.74, *P* < 0.001, respectively). Second, Bland–Altman analyses revealed no substantial bias between RF using 2D multi-venc and RF using standard 2D flow mapping (1.3 ± 5.4%) nor between RF using 4D flow and RF using standard 2D flow mapping (0.3 ± 5.4%). Third, the intra-observer and inter-observer reproducibility of standard 2D flow and 2D multi-venc measurements is excellent, and the reproducibility of 4D flow measurements is good. Fourth, no significant reclassification of PVR severity occurred between the different CMR flow acquisition techniques, and there was a comparable reclassification between the assessment of PVR with the individual CMR techniques and TTE. These findings underline the strong potential of 2D multi-venc and 4D flow mapping in the assessment of PVR after TAVR.

Even though the incidence of PVR is decreasing with newer-generation TAVR devices and enhanced operator skills,^26^ the risk of PVR is still considered an important shortcoming of TAVR. Given the negative impact on patient outcomes, both in terms of morbidity and mortality, every effort should be made to reduce the risk of PVR.^3,11,27^ Accurate grading of PVR is therefore essential.

Multiple imaging modalities, such as TTE and angiography, are being used for the assessment of PVR after TAVR. TTE is widely available, inexpensive, and easy to use. However, TTE has been shown to underestimate the degree of PVR when compared with CMR.^28–32^ Angiography using the visual Sellers’ method is also widely available but lacks accuracy in quantifying PVR.^31,32^

CMR is considered an accurate and reproducible technique in the assessment of PVR and is acknowledged as the gold standard for measurements of LV volume, mass, and ejection fraction.^33,34^ CMR has a high contrast-to-noise ratio and a high spatial and temporal resolution, allows quantification in unlimited imaging planes, and is not dependent on contrast administration. In patients in whom PVR assessment by TTE is equivocal or when clinical parameters are not in accordance with the degree of PVR measured with TTE, CMR can be considered as quantitative modality. However, compared with TTE, CMR is more expensive, is not a bedside tool, has longer acquisition and post-processing times, and can be challenging in patients with cardiac arrythmia and claustrophobia.

Two-dimensional PC venc using through-plane velocity quantification is the most frequently adopted CMR technique for PVR assessment, in which one venc value is used. This single-venc value is generally set at a high venc of ≥180 cm/s, providing accurate measurements of the FV but with lower accuracy in RV (low-flow volume) measurement, due to the lower signal-to-noise ratio.^35^

The 2D flow acquisitions are performed during successive end-expiratory breath-holds. In the frail TAVR population with frequent pulmonary comorbidities, these serial breath-holds could be exhausting, increasing the risk of suboptimal imaging quality, and hence lead to inaccurate results. Alternatively, non–breath-hold acquisitions can be performed, with the expense of significantly longer acquisition times (1.5 min vs. 10–15 s in breath-hold).

Two emerging techniques in the field of CMR flow assessment are 2D multi-venc and 4D flow mapping, which may overcome these issues. Two-dimensional multi-venc has the advantage of integrating three different venc values into a single flow curve, therefore only necessitating one breath-hold acquisition. With this broad range of venc values captured, both high-flow and low-flow volume assessments can be accurately performed.

Four-dimensional flow mapping has several advantages: first, it is effective in providing a comprehensive visualization of the blood flow with proven effectiveness in accurately measuring velocity in all spatial directions. Comprehensive visualization of paravalvular regurgitant jets is of importance when considering percutaneous PVR closure by a vascular plug. Second, 4D flow mapping does not require specialized cardiac anatomy knowledge or specific imaging planes for acquisition, since it can cover the whole heart.^36^ The continuous presence of a CMR laboratory technician could therefore be reduced. Third, the analysis plane can be set anywhere within the acquisition area retrospectively, which reduces planning effort, allows standardization of acquisition, and facilitates post-processing. Last, 4D flow mapping acquisitions do not depend on breath-holds, allowing the patient to breathe freely. Especially in the TAVR population, this can be of great benefit. Besides the numerous advantages of the 4D flow mapping acquisition, important disadvantages are the longer acquisition time of ∼3 min and the risk of breathing artefacts. In addition, adequate 4D flow planning and acquisition has a learning curve and requires strong collaboration between cardiac imagers and their technicians during the implementation phase.

### Future perspectives

The annual number of patients treated by TAVR is still rising,^26^ with an inherent increase in cases in which the degree of PVR as assessed with TTE can be debatable. In these cases, CMR can be the designated imaging modality to provide clarity regarding PVR severity. The advantages of the novel CMR techniques addressed in this study can be considered in the decision of which acquisition technique to use. Given the high correlation with the standard 2D flow mapping technique and the acceptable inter-observer variability, 4D flow mapping appears to be a valuable alternative to standard 2D flow mapping, limiting the risk of inadequate plane selection and reducing the need for the continuous presence of a CMR laboratory technician.

### Limitations

This study has some limitations. First, the number of patients in this study is relatively low, and not all patients underwent all three different flow assessment techniques due to the extensive scan protocol. However, we do believe that the results of this study would not change even if a larger sample size is used, given the strong correlation between the CMR sequences and the high reproducibility of these sequences. Second, the 4D flow imaging plane was visually set at the same level as for the standard 2D and 2D multi-venc sequences. Therefore, it is unknown whether differences in flow volumes can be attributed to differences in plane selection. Third, since the number of patients with ≥moderate PVR is limited, conclusions on the reclassification between the CMR modalities for this patient category can not be drawn. Fourth, this study was performed solely with the Abbott Portico valve, precluding the direct translation of these results to other types of TAVR devices. However, since the focus of this study was to compare the three different CMR flow mapping acquisitions for PVR assessment, rather than describing the CMR–RF of different valve types, the aforementioned point is of less importance. Fifth, the 4D flow mapping sequence uses only a single (high) venc value, as opposed to the standard 2D flow and 2D multi-venc flow sequences, thereby potentially limiting the accuracy of the RVs.

## Conclusion

Two-dimensional multi-venc and 4D flow mapping are two novel techniques that have a high correlation with standard 2D flow mapping in the quantification of PVR after TAVR. The high acquisition speed of the 2D multi-venc sequence and the free-breathing acquisition with retrospective plane selection with 4D flow mapping provide useful advantages, especially in the frail TAVR population. Given these practical advantages, the use of these techniques should be considered.

## Data Availability

The data underlying this article are available from the corresponding author on reasonable request.
